# Systematic analysis of the effects of genetic variants on chromatin accessibility to decipher functional variants in non-coding regions

**DOI:** 10.3389/fonc.2022.1035855

**Published:** 2022-10-18

**Authors:** Dongyang Wang, Xiaohong Wu, Guanghui Jiang, Jianye Yang, Zhanhui Yu, Yanbo Yang, Wenqian Yang, Xiaohui Niu, Ke Tang, Jing Gong

**Affiliations:** ^1^ Hubei Key Laboratory of Agricultural Bioinformatics, College of Informatics, Huazhong Agricultural University, Wuhan, China; ^2^ Department of Biochemistry and Molecular Biology, Tongji Medical College, Huazhong University of Science and Technology, Wuhan, China; ^3^ College of Biomedicine and Health, Huazhong Agricultural University, Wuhan, China

**Keywords:** Single nucleotide polymorphism, Chromatin accessibility, Quantitative trait locus, caQTL, Breast cancer

## Abstract

Genome-wide association study (GWAS) has identified thousands of single nucleotide polymorphisms (SNPs) associated with complex diseases and traits. However, deciphering the functions of these SNPs still faces challenges. Recent studies have shown that SNPs could alter chromatin accessibility and result in differences in tumor susceptibility between individuals. Therefore, systematically analyzing the effects of SNPs on chromatin accessibility could help decipher the functions of SNPs, especially those in non-coding regions. Using data from The Cancer Genome Atlas (TCGA), chromatin accessibility quantitative trait locus (caQTL) analysis was conducted to estimate the associations between genetic variants and chromatin accessibility. We analyzed caQTLs in 23 human cancer types and identified 9,478 caQTLs in breast carcinoma (BRCA). In BRCA, these caQTLs tend to alter the binding affinity of transcription factors, and open chromatin regions regulated by these caQTLs are enriched in regulatory elements. By integrating with eQTL data, we identified 141 caQTLs showing a strong signal for colocalization with eQTLs. We also identified 173 caQTLs in genome-wide association studies (GWAS) loci and inferred several possible target genes of these caQTLs. By performing survival analysis, we found that ~10% caQTLs potentially influence the prognosis of patients. To facilitate access to relevant data, we developed a user-friendly data portal, BCaQTL (http://gong_lab.hzau.edu.cn/caqtl_database), for data searching and downloading. Our work may facilitate fine-map regulatory mechanisms underlying risk loci of cancer and discover the biomarkers or therapeutic targets for cancer prognosis. The BCaQTL database will be an important resource for genetic and epigenetic studies.

## Introduction

As the most common type of genetic variation, single nucleotide polymorphism (SNP) plays a vital role in complex human diseases. Genome-wide association studies (GWAS) have identified thousands of SNPs associated with human traits or diseases (1). However, the vast majority of them are located inside the non-coding regions (2), and their functions remain largely unknown. Many studies have emphasized the important role of non-coding SNPs in multiple regulatory processes, including histone modifications (3) and TF binding (4). For example, SNPs within CTCF binding sites could disrupt the binding site, thus altering the loop formation and chromatin topology, ultimately affecting target gene expression (5). Studies have also shown that SNPs could affect histone functions in human liver (6), lupus patient lymphoblastoid cell lines (7), and obesity-related traits (8), indicating that these SNPs may affect histone modification or histone binding, which indirectly changes the chromatin accessibility.

As one of the high-throughput sequencing, ATAC-seq was used to identify open chromatin regions which usually represent cis-regulatory elements (9, 10). Increasing evidence has shown that chromatin accessibility is crucial for gene expression regulation in specific cells and multiple biological processes, and abnormal chromatin accessibilities have been observed in various human diseases, especially cancers (11–13). Quantitative trait locus (QTL) analysis is a statistical method that links genotypic data with phenotypic data and has been demonstrated as a powerful tool for interpreting the SNP function on molecular traits such as gene expression and chromatin accessibility (11, 14–18). Recent studies have shown that SNPs could alter chromatin accessibility and result in differences in tumor susceptibility between individuals. Chromatin accessibility quantitative trait loci (caQTL) analyses have been performed in some human tissues and diseases (11, 18, 19). However, systematic analyses of the effects of genetic variation on chromatin accessibility in human cancers have rarely been conducted.

The Cancer Genome Atlas (TCGA) (20) generated a large amount of omics data, including genotype data, clinical information, and ATAC-seq data (20). The rich data from TCGA make it possible to systematically identify genetic variants altering chromatin accessibility in human cancers. In this study, to gain insight into the genetically regulated chromatin accessibility in human cancers, we performed caQTL analysis by integrating matched genotypes and ATAC-seq data. In addition, we further characterized the functional mechanisms of these caQTL SNPs and their potential clinical application by integrating caQTL data with other omics and clinical data.

## Materials and methods

### Genotype data processing

We obtained the genotype matrix of 898,620 SNPs detected using the Affymetrix SNP 6.0 array from the TCGA data portal (20) (https://tcga-data.nci.nih.gov/tcga/). For better performance in QTL analysis, we imputed autosomal variants for all samples in 23 types of cancer using IMPUTE2 (21) (1000 Genomes Phase 3 (22) as the reference panel). The imputation was performed in the two-step procedure provided by IMPUTE2. After imputation, we performed quality control (23) to select high-quality SNPs with: (i) imputation confidence score (INFO) ≥ 0.4, (ii) minor allele frequency (MAF) ≥ 5%, (iii) missing rate < 5 %, and (iv) Hardy-Weinberg Equilibrium *p* > 1e-6 (estimated by Hardy-Weinberg R package). To eliminate the impact of population structure on chromatin accessibility, we used smartpca in the EIGENSOFT program to generate the principal component factors. We included the first five principal components in the covariates.

### ATAC-seq data processing

We obtained all the ATAC-seq data from TCGA (https://tcga-data.nci.nih.gov/tcga/), containing ATAC-seq bam files of 410 tumor samples from 404 TCGA donors across 23 cancer types. The processing strategy described in Corces et al. (24) was used to process ATAC-seq data. To obtain open chromatin regions, the “call peak” command in MACS2 with parameters “–shift -75 –extsize 150 –nomodel –call-summits –nolambda –keep-dup all -p 0.01” was used to perform peak calling. Then the peak summits were extended by 250 bp on both sides, resulting in a set of fix-width peaks of 501 bp width. Peaks that extend beyond the length of chromosomes or overlap with the DAC Exclusion List Regions from ENCODE were excluded in the downstream analysis.

We used an iterative removal procedure (24) to identify independent peak calls in each sample. For each sample, we first sorted the peaks in descending order of the peak scores (the “pValue” column). Next, we kept the most significant peak and removed any peak which overlapped with that peak. Finally, we sort the remaining peaks and repeat the previous operation until no peaks overlap with each other.

Since the score calculated by MACS2 varies when samples have different read depth or quality, we should normalize the scores of the significance of peaks across samples and cancer types to enable the comparison between samples and between different cancer types. The peak score in each sample was first converted to score per million (SPM). Then, to obtain independent peak sets in each cancer type, we combined peak sets from all the samples in that cancer type. Next, we used the same iterative removal procedure to obtain independent peaks. We only kept peaks that: (i) were observed in over two samples, (ii) have an SPM ≥ 5, (iii) did not span the gap region, and (iv) were on the autosomes. These SPM matrices were used in caQTL mapping and downstream analysis.

Finally, to remove the hidden batch effects or other confounders in the chromatin accessibility data, the PEER R package was used to generate the PEER factors from chromatin accessibility data to minimize the batch effect. Reported that PEER factors equivalent to 25% of the sample size were selected to avoid the batch effect of the chromatin accessibility data (25).

### The enrichment of chromatin state in ATAC-peaks

We obtained the data on chromatin states from the Roadmap Epigenomics Consortium. We combined related chromatin states and redefined the states as follow: (i) 1_TssA, 2_TssFlnk, 3_TssFlnkU, 4_TssFlnkD, and 14_TssBiv to promoter, (ii) 5_Tx and 6_TxWk to transcribed, (iii) 7_EnhG1, 8_EnhG2, 9_EnhA1, 10_EnhA2, 11_EnhWk, and 15_EnhBiv to enhancer, (iv) 16_ReprPC and 17_ReprPCWk to polycomb, (v) 12_ZNF/Rpts to ZNF repeats, (vi) 13_Het to heterochromatin and 18_Quies to quiescent. We calculated the coverage of each ATAC-peak for each chromatin state using the BEDTools “coverage” command. The peak was assigned to the chromatin state with the highest coverage, except for quiescent. Only when the peak has a coverage of 100% with quiescent will it be assigned to quiescent. Otherwise, that peak will be assigned to the chromatin state with the second-highest coverage. Background peaks were selected randomly by the bedtools shuffle command.

### Selection of expressed transcription factors in cancer

We downloaded all of the 838 non-redundant transcription factors (TF) binding motifs for vertebrates from JASPAR CORE 2022 (26) (https://jaspar.genereg.net/downloads/) and restricted to TFs expressed in cancer. To do this, we obtained the expression data of all cancer types from TCGA and TFs with a median TPM > 1 marked as expressed in that cancer type. Only the expressed TFs were included in TF enrichment and motif disruption analyses.

### Enrichment of transcription factor binding sites in ATAC-peaks and caQTL peaks

Using the non-redundant expressed TFs and the Analysis of Motif Enrichment (AME) software (27), we tested for the TF enrichment in ATAC-peaks. To do this, we first extracted the sequence of the peaks from the reference genome using BEDTools. The “–control generated the control sequences for the enrichment test –shuffle–” command of AME. The full arguments we used was “–control –shuffle– –kmer 2 –scoring max –hit-lo-fraction 0.75”. Motifs with an E-value < 1e-100 were classified as significantly enriched.

### caQTL mapping

caQTL was mapped using the MatrixEQTL (28) R package. We chose the linear model to quantify the statistical association of chromatin accessibility with the genotypes of variants in each cancer type. Cis-caQTL mapping was performed in a window of 1 Mb, and the significant associations were defined if FDR was smaller than 0.05. Covariates were used to eliminate the hidden cofounders. In addition to the PEER and principal component factors, we also included essential clinical information, including age, gender, and tumor stage, to avoid the potential impact of clinical status on caQTL mapping.

### Colocalization of caQTLs and eQTLs

Colocalization analysis could find the causal variation that caQTLs and eQTLs may share. To do this, we used the Bayesian test for colocalization implemented in the coloc R package to assess the probability that cis-caQTLs and cis-eQTLs shared the same causal variant. The cis-eQTL data used in this analysis came from our previous research (14). We adopt a two-step procedure for colocalization. The first step is to identify the QTL pairs which may share a causal variant for the test and then perform the Bayesian test for colocalization.

To identify the QTL pairs for the colocalization test, we extracted the lead caQTL of the caQTL peak for each caQTL peak. The eQTL gene correlated with this lead caQTL was tested for colocalization. If multiple genes are linked with the same lead caQTL, we first obtained the lead eQTL SNP of each eQTL gene, and then we calculated the LD between the lead eQTL SNP and the lead caQTL SNP. Only the eQTL genes whose lead eQTL SNP has the highest LD with the lead caQTL SNP were retained. For each caQTL peak, this workflow helped us obtain a peak-gene pair that showed an association with the same SNP. Then, we applied the Bayesian model implemented in the coloc R package to all peak-gene pairs. Colocalization results with a PP4 > 0.8 were regarded as solid colocalization evidence.

### Mediation analysis

Using the colocalized peak-gene pairs, we applied model-based causal meditation analysis implemented in the mediation R package to investigate whether the effect of a cis-eQTL SNP is mediated by influencing chromatin accessibility. To do this, we extract the chromatin accessibility and gene expression data of all matched samples for each colocalized pair. We fitted two statistical models to estimate the effect direction of the SNP. In the mediator model, we hypothesize chromatin accessibility as the mediator and gene expression as the outcome. Moreover, we reverse the mediator and the outcome in the outcome model. The average causal mediation effects (ACME) calculated by coloc were used to characterize the mediation effects in a specific model. In addition, we used the nonparametric bootstrap (resample 1,000 times) instead of the quasi-Bayesian Monte Carlo simulation for variance estimation.

### Identification of GWAS-related caQTLs

We obtained the latest release of the NHGRI-EBI GWAS catalog (1). For this data, we first extracted all the single-variant associations. We extracted each GWAS SNP’s alleles from the dbSNP (build 155) dataset and removed all the non-bi-allelic variants for LD calculation. Next, we extracted the LD region of each SNP using a threshold of R^2^ > 0.5 calculated by LDlink (https://ldlink.nci.nih.gov/). The caQTL SNPs falling into the LD regions were identified as GWAS-related caQTLs. LocusZoom (29) was used for visualizing specific loci.

### Transcription factor binding sites disrupted by caQTL SNPs

To find all TF binding sites potentially disrupted by caQTLs, we selected all the caQTL SNPs falling in the caQTL peaks using the BEDTools “intersect” command. For both alleles of each caQTL, we extracted the DNA sequence containing the variant and extended it by 30 bp on both sides. We then scanned these sequences for matches of the motifs using the Find Individual Motif Occurrences (FIMO) software (30). Only the most significant match per allele and the match that overlapped with the caQTL position was retained. The log ratio of *p*-values defined as lg(p_weak) - lg(p_strong) was used for quantifying the difference in motif match between alleles (31), where the p_weak and the p_strong stand for the *p*-values for the alleles with the weaker and stronger match, separately.

### Identification of survival-related caQTLs

To identify caQTLs associated with disease prognosis, we obtained clinical information, including the survival time of all donors from TCGA. We divided all the donors into groups based on the SNP genotype and used the Log-Rank test to test the difference in survival time between these groups. There were three models that we used in this study. In the additive model, we divided the donors into three groups based on the three genotypes of each SNP and tested the difference in survival time among the three groups. In the dominant model, the heterozygous donors (Aa) and the homozygous carriers of the minor allele (aa) were merged into one group and tested against the other. In the recessive model, the heterozygous donors (Aa) and the homozygous carriers of the major allele (AA) were merged into one group and tested against the other group. The Kaplan–Meier plot was used for visualization.

## Results

### Open chromatin region related to regulatory elements

**Figure 1:**
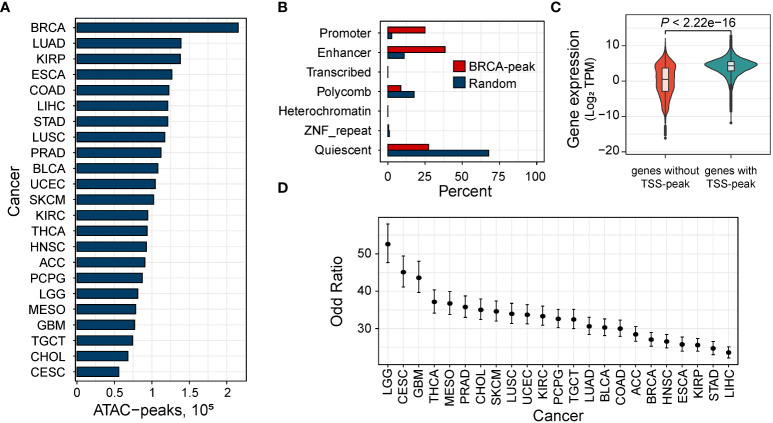
Open chromatin region related to regulatory elements. **(A)** Distribution of the number of the ATAC-peaks in 23 human cancers. BRCA, breast invasive carcinoma; KIRP, kidney renal papillary cell carcinoma; PRAD, prostate adenocarcinoma; COAD, colon adenocarcinoma; LUAD, lung adenocarcinoma; STAD, stomach adenocarcinoma; ESCA, esophageal carcinoma; LIHC, liver hepatocellular carcinoma; KIRC, kidney renal clear cell carcinoma; LUSC, lung squamous cell carcinoma; THCA, thyroid carcinoma; LGG, low-grade glioma; BLCA, bladder urothelial carcinoma; HNSC, head and neck squamous cell carcinoma; PCPG, pheochromocytoma and paraganglioma; TGCT, testicular germ cell tumors; ACC, adrenocortical carcinoma; MESO, mesothelioma; SKCM, skin cutaneous melanoma; UCEC, uterine corpus endometrial carcinoma; CHOL, cholangiocarcinoma; CESC, cervical squamous cell carcinoma; GBM, glioblastoma multiforme. **(B)** Percent of ATAC-peaks of breast carcinoma by chromatin state in breast tissue from the Roadmap Epigenomics Project. All peaks, red; randomly selected genomic regions. **(C)** Fisher’s test shows that expressed genes (median TPM > 1) are more likely to have an ATAC-peak overlapping the TSS. Error bars indicate 95% confidence intervals. **(D)** Comparison of the expression between genes expressed in BRCA with and without an ATAC-peak overlapping the TSS.

We downloaded the ATAC-seq data of 410 samples derived from 404 tumor donors across 23 cancer types from The Cancer Genome Atlas (TCGA). We then performed peak calling using MACS2 (https://github.com/macs3-project/MACS) to get open chromatin regions. By adopting the strategy described by Corces et al. (24), we normalized each peak’s score for downstream analysis. As a result, we obtained a collection of open chromatin regions with a fixed width. The number of these peaks ranges from 55,979 (in cervical squamous cell carcinoma and endocervical adenocarcinoma, CESC) to 215,354 (in invasive breast carcinoma, BRCA) with an average of 105,307 peaks per cancer type (**Figure 1A**).

To explore the regulatory function of these ATAC-peak, we mapped our ATAC-peaks to chromatin states of matched tissues obtained from the Roadmap Epigenomics Project (32). As expected, compared to peaks randomly selected, accessible peaks were enriched in enhancers and promoters (**Figure 1B** and **Supplementary Figure S1**), indicating that accessible regions were primarily located in regulatory regions. We checked whether genes with ATAC-peaks at the transcription start site (TSS) were more likely to be expressed (median transcript per million (TPM) > 1) than those without peaks at the TSS. In all the cancer types, we found that expressed genes were more likely to have an ATAC-peak overlapping the TSS (minimum OR = 23.57 in hepatocellular liver carcinoma, **Figure 1C**) than those not expressed. Then for expressed genes, on the other hand, genes with ATAC-peaks overlapping their TSS tend to have a higher expression than those without TSS peaks (**Figure 1D** and **Supplementary Figure S2**).

Then we tested for the enrichment of the transcription factor (TF) binding sites in these ATAC-peaks. We found 374 TFs significantly enriched (Evalue < 1e-100) in all of the 23 cancer types, including 125 oncogenes on the list of oncogenes provided by the Network of Cancer Genes (33) (NCG v7.0), which indicated that ATAC-peaks reflect active regulatory elements in cancer tissues.

### Genetic variants associated with chromatin accessibility

**Figure 2:**
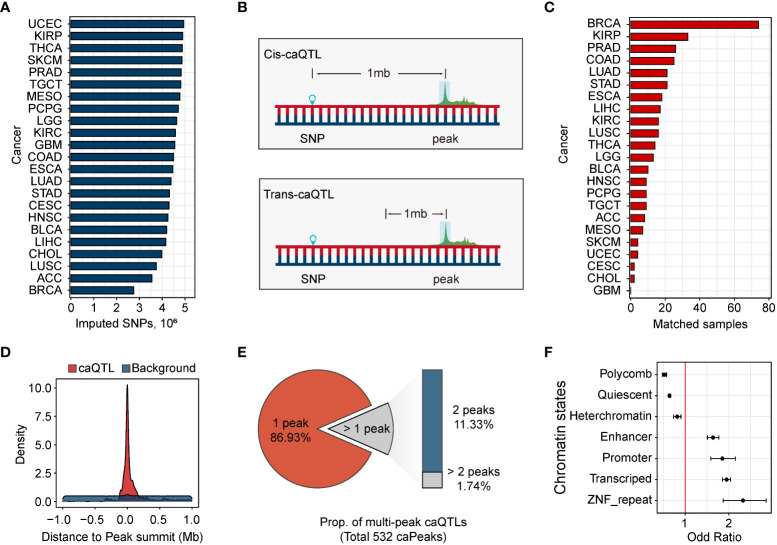
Genetic variants associated with chromatin accessibility. **(A)** Distribution of the number of the ATAC-peaks in 23 human cancers. **(B)** Cis-caQTLs are identified using the variants within 1 Mb of ATAC-peaks, and caQTLs outside the window are defined as trans-caQTLs. **(C)** The number of samples with matched ATAC-seq data and SNP genotype data. There is no matched sample in GBM. **(D)** Distribution of the distance between caQTL SNPs and their related caQTL peaks. Distance distribution between caQTL SNPs and caQTL peak, red. Distance distribution between all tested variants and their tested ATAC-peaks, blue. **(E)** Distribution of the number of single-peak caQTLs and multi-peak caQTLs. **(F)** Enrichment of caQTL SNPs in breast chromatin states. Error bars represent 95% confidence intervals.

Using imputed genetic variants (**Figure 2A**), we mapped caQTLs using the linear model implemented in MatrixEQTL (28). In caQTL mapping, a window of 1 Mb was used to distinguish cis-caQTL from trans-caQTLs (**Figure 2B**). To correct for hidden confounders, we introduced covariates from the population, chromatin accessibility, and clinical information (Methods). Using the threshold of false discovery rate (FDR) < 0.05, we identified 8,194 SNPs associated with 532 ATAC-peaks in BRCA, accounting for 0.25% of all the ATAC-peaks and 0.30% of the SNPs in BRCA. All these caQTLs were located within the 1 Mb window of their associated peaks (cis-caQTLs). However, in other cancers, none of the caQTLs passed our threshold. Considering that the second-highest sample size of cancers is 33 in the kidney renal papillary cell carcinoma (KIRP) (**Figure 2C**), we attribute this result to the insufficient sample size. Hence, we focused on the cis-caQTLs identified in BRCA.

Consistent with previous reports (11, 18, 33), caQTLs tend to be close to their related caQTL peaks. A substantial portion of cis-caQTLs (n = 6,757, 71.3%) was located within the 0.1-Mbp region of their related caQTL peaks (**Figure 2D**), suggesting the regulation of SNP on chromatin accessibility was mainly local. The majority (86.9%) of the caQTLs were assigned to a single caQTL peak, while other caQTLs were assigned to multiple caQTL peaks (**Figure 2E**). As expected, caQTL SNPs were significantly enriched in promoters (OR = 1.85), enhancers (OR = 1.64), and transcribed regions (OR = 1.95), and depleted in polycomb (OR = 0.52), heterochromatin (OR = 0.81), and Quiescent (OR = 0.64) states (**Figure 2F**).

### Several caQTLs could also affect gene expression

**Figure 3:**
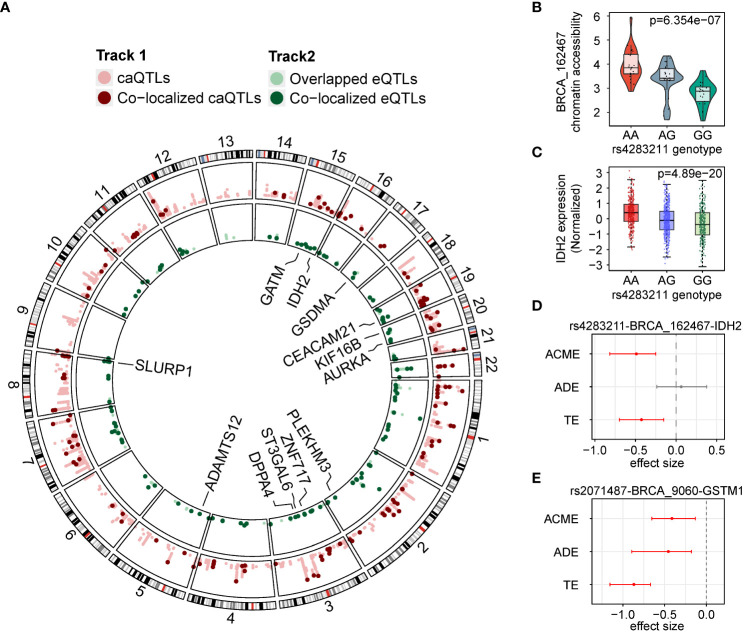
caQTLs overlapped with eQTLs. **(A)** Circos plot of colocalized caQTLs and eQTLs. Outer track lighter red, caQTL SNPs. Outer track darker red dots colocalized caQTL SNPs. Inner track lighter green, eQTL variants overlapped with caQTL SNPs. Inner track darker green dots colocalized eQTL variants. **(B)** Distribution of chromatin accessibility of BRCA_162467 in groups with different genotypes of rs4283211. **(C)** Distribution of expression of *IDH2* in groups with three different genotypes of rs4283211. **(D)** Effect sizes in mediation analysis on the rs4283211-BRCA_162467-*IDH2* pair. ACME, average causal mediation effects; ADE, average direct effects and TE, total effect. **(E)** Effect sizes in mediation analysis on the rs2071487-BRCA_9060-*GSTM1* pair.

We compared caQTLs to eQTLs identified previously (14). We found that 3,406 (41.6%) of the caQTL SNPs were also observed in eQTLs, contained in 7,576 SNP-gene- ATAC peak pairs. According to the peak-gene linking analysis in Corces et al. (24), 1,543 out of 7,576 pairs (1,299 unique SNPs) were found to contain SNPs that affect the same genes by the corresponding caQTLs and eQTLs, indicating that changes in chromatin accessibility may also affect gene expression. We performed a Bayesian colocalization analysis to identify the potential causal SNPs shared by caQTLs and eQTLs (35), and identified 141 SNPs with strong evidence for having shared genetic effects (posterior probability > 0.8, **Figure 3A**). Among these colocalized caQTL SNPs, 13 SNPs were also related to the expression of genes reported to be potential driver genes in cancer. For example, the G allele (allele frequency (AF) = 0.547) of rs4283211 was predicted to downgrade the chromatin accessibility of BRCA_162467 (chr15:90647785-90648286, *p* = 6.35e-7, **Figure 3B**). This allele was also predicted to cause a decrease in the expression of gene *IDH2* (*p* = 4.89e-20, **Figure 3C**). *IDH2* was a widely reported driver gene in multiple cancers (36–38). According to the mutation distribution data for *IDH2* from the COSMIC database (38, 39), the number of mutants of A-to-G and G-to-A together accounted for 77.95% of the total mutants, indicating that the regulation of variants to *IDH2* may have preference.

To assess the evidence that chromatin accessibility mediates the effect of a SNP on local gene expression, we performed mediation analysis on our 141 colocalized SNP-peak-gene pairs. Under a threshold of adjusted *p* < 0.05, we observed 19 SNP-gene-peak pairs showing evidence of mediating effect (total effect *p* < 0.05 and mediation *p* < 0.05), and four of them were in the oncogenes list mentioned before. It is worth noting that the rs4283211-BRCA_162467-*IDH2* pair has a significant mediating effect (*p* < 0.001, **Figure 3D**) but showed no signs of direct impact (*p* = 0.65), which means that the effect of SNP on gene expression is entirely through chromatin accessibility. Another example was the pair rs2071487-BRCA_9060-*GSTM1* (**Figure 3E**), which showed significance in both mediating effects (*p* = 0.004) and significant direct effect (*p* < 0.001), indicating that the impact of this SNP on the expression of *GSTM1* may be exerted through the accessibility changing of BRCA_9060 (chr1:110230129-110230630).

### caQTLs disrupted the binding sites of TFs

**Figure 4:**
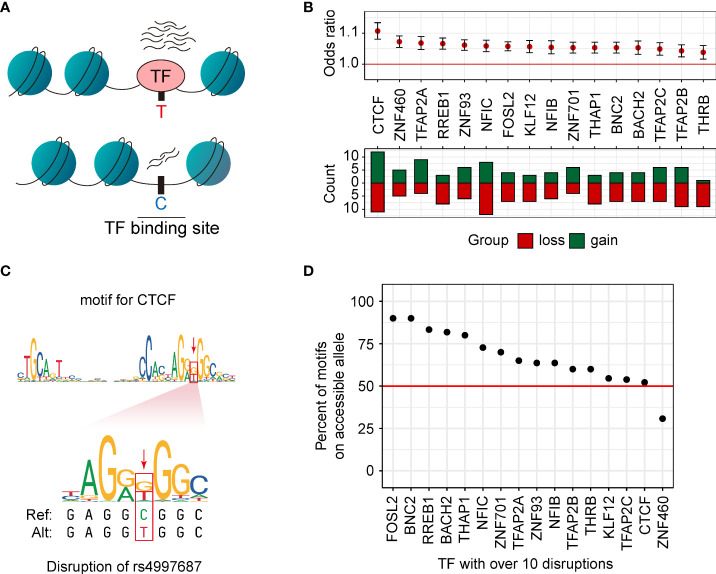
caQTLs disrupted the binding sites of TFs. **(A)** Motif disruption analysis was performed using the caQTL SNPs in their related caQTL peaks. **(B)** Disruption of motifs is significantly associated with the caQTL status. Loss, the reference alleles match the motif better. Gain, the alternative alleles match the motif better. **(C)** An example of the disruption of rs4997687 in the motif of *CTCF*. **(D)** The proportion of the disrupted motifs for which the allele with higher chromatin accessibility matched the motif better. Only the 16 TFs with more than ten disruptions are shown. The red line represents the average percent across the 16 TFs.

Altering chromatin accessibility by affecting the transcription factor binding sites (TFBS) is an important regulatory pathway of genetic variants (18, 40, 41) (**Figure 4A**). In our caQTL results, 187 caQTL SNPs were located in 104 caQTL peaks. Among these in-peak caQTLs, 172 (92.0%) caQTL SNPs could change the binding affinity of TFs (Methods). Of the 104 caQTL peaks containing the related caQTL SNPs, 96 caQTL peaks contained at least one motif-disrupting caQTL SNP, while 40 of these caQTL peaks contained more than one motif-disrupting caQTL SNP. 16 motifs were disrupted by ten or more caQTL SNPs. Almost all these motifs (15 of 16) were significantly related to caQTL status (OR > 1, *p* < 4.6e-4, **Figure 4B**), including motifs of five oncogenes including *CTCF*, *RREB1*, *TFAP2A*, *BNC2*, and *BACH2*. According to previous reports, CTCF induced the expression of *Nm23-H1* and was related to cell migrations in MDA-MB-231 cell lines (42). In our study, the binding sites of *CTCF* were affected by rs4997687 (**Figure 4C**), suggesting that *CTCF* may be an important mediator for several caQTLs exerting their functions. We then investigated whether TFs are more likely to bind the more accessible allele. TFs are theoretically more likely to bind the alleles with higher accessibility (31). In our results, more than half of the 406 motifs of 377 expressed TFs (59.9%) bind the alleles with more accessibility better. When restricting to the TFs with more than ten disruptions, similar results (60.0%) were observed, and this percentage varies among different TFs (**Figure 4D**). The *FOSL2* and *BNC2* almost only bind the alleles with higher accessibility. Together, these results reveal that caQTL SNP could exert its function by affecting the TF binding but having TF specificity.

### Possible regulatory caQTL SNPs and target genes at GWAS loci

**Figure 5:**
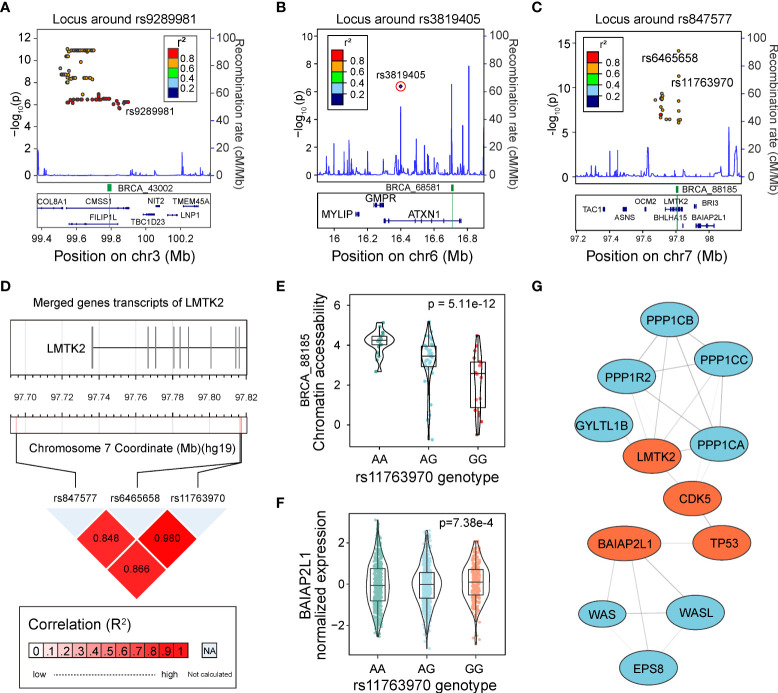
Identification of regulatory caQTLs at GWAS loci. **(A)** Locus plot shows the LD region of rs9289981. caQTL is marked in purple rhombus, and the caQTL peak is highlighted in green. **(B)** The locus plot shows the LD region of rs3819405. caQTL is marked in a circled purple rhombus, and the caQTL peak is highlighted in green. **(C)** The locus plot shows the LD region of rs847577. The caQTL rs11763970 and rs6465658 are marked, and the caQTL peak is highlighted in green. **(D)** The linkage disequilibrium (LD) of the GWAS tag SNP rs847577, caQTL variant rs11763970, and caQTL variant rs6465658. **(E)** Distribution of chromatin accessibility of BRCA_88185 in groups with different genotypes of rs11763970. **(F)** Distribution of expression of *BAIAP2L1* in groups with three different genotypes of rs11763970. **(G)** Protein-protein network of gene *BAIAP2L1* and *LMTK2*, both of which interact with *TP53*. Genes involved in the interaction are highlighted in red.

To identify the potential causal caQTLs and genes located in GWAS loci, we performed a linkage disequilibrium (LD)-based-colocalization analysis on our caQTLs and GWAS SNPs downloaded from the GWAS Catalog (1, 43). Using GWAS variants of breast carcinoma and a threshold of R^2^ > 0.5, we identified 173 GWAS-related caQTLs mapped to three GWAS loci. Previous studies have not reported clear causal SNPs and genes for these three GWAS loci. Among these caQTLs, only two caQTL SNPs, rs9289981 (**Figure 5A**) and rs3819405 (**Figure 5B**), directly overlapped with GWAS SNPs. However, rs9289981 was not the most significant caQTL mapped to peak BRCA_43002.

Another example was rs11763970, the second significant GWAS-related caQTLs in the LD block of the GWAS SNP rs847577 mapped to *LMTK2* after rs6465658 (**Figure 5C**). rs6465658 was also a GWAS SNP for prostate cancer and a prostate cancer eQTL variant for gene *BHLHA15* (*p* = 4.02e-4) but showed no significant effect on gene expression in breast cancer. The three SNPs showed high LD with each other (R^2^ > 0.8, **Figure 5D**). Although rs11763970 was not the most significant caQTL in that LD block, it showed a significant association with the gene *BAIAP2L1* in eQTLs of BRCA (**Figures 5E, F**). Furthermore, according to the evidence from the STRING database (44), *BAIAP2L1* has evidence for protein-protein interaction with *TP53* (**Figure 5G**), indicating the potential regulation among variants, accessible chromatin, and gene expression in human cancer.

### caQTLs related to patient survival time

**Figure 6:**
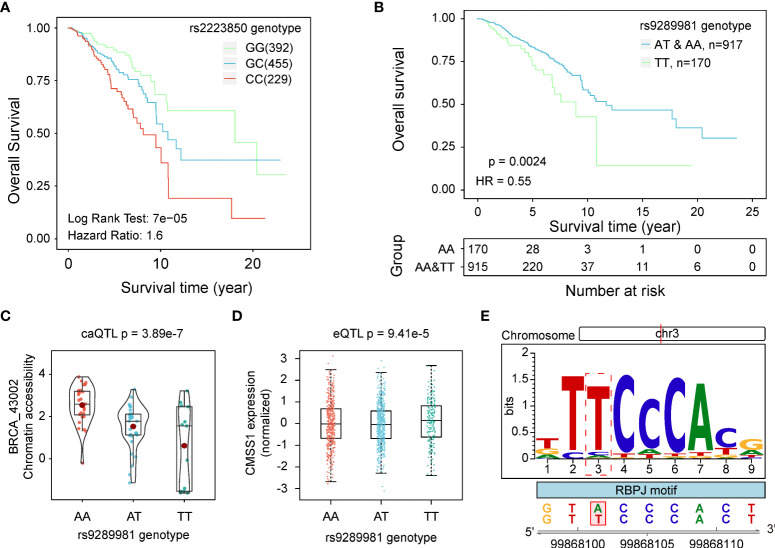
caQTLs related to survival time. **(A)** KM curves show the difference in survival time of patients with different genotypes of rs2223850. **(B)** KM curves show the difference in survival time of patients with different genotypes of rs9289981 in the dominant model. Patients in the TT genotype group showed a poor prognosis. **(C)** Distribution of chromatin accessibility of BRCA_43002 in groups with different genotypes of rs9289981. **(D)** Distribution of expression of *CMSS1* in groups with three different genotypes of rs9289981. **(E)** Potential disruption of rs9289981 on the binding site of *RBPJ*.

In our previous studies, we have identified many survival-related QTLs in different molecular traits. To determine whether SNPs related to chromatin accessibility are associated with patient survival time, we performed survival analysis on the caQTL SNPs we identified. We assessed the significance of the difference in survival time among three genotype groups (additive genetic model) and identified 730 caQTL SNPs significantly (*p* < 0.05) associated with survival time. For example, the G-to-C mutation of rs2223850 was significantly correlated with a worse prognosis (*p* < 0.001, HR = 1.59 [1.29, 1.98], **Figure 6A**). During the analysis, we noticed that the correlation between genotypes and survival time showed deviations from linearity in some of the caQTL SNPs. Specifically, the survival time observed for the heterozygous individuals was different from the expected values based on the observation of the homozygous individuals. Thus, we separately performed survival analysis with dominant and recessive models for a better understanding of the impact of genotypes on patient survival time. Under new models, we identified 483 and 553 more survival-related caQTLs, respectively, increasing the number of survival-related caQTLs to 1,069. For each survival-caQTL, we also extracted the genes linked to the caQTL-related peaks using the peak-gene link data (24) and performed gene-base survival analyses. As a result, we totally found 122 SNP-peak pairs (116 unique caSNPs), in which both SNPs and their peak-linked genes were significantly associated with the patient survival time (*Log Rank P* < 0.05). An example was the caQTL rs9289981, a GWAS-related caQTL significantly related to the overall survival time of BRCA patients. Individuals with T/T genotype exhibited worse prognosis than those with A/T and A/A (*p* < 0.001, HR = 0.55 [0.37, 0.81], **Figure 6B**). This caQTL was also a GWAS-related BRCA caQTL and the eQTL of *CMSS1*, with its T allele associated with the increase in the chromatin accessibility of caQTL peak BRCA_43002 (**Figure 6C**) and the decrease in expression of *CMSS1* (**Figure 6D**). Furthermore, TF binding prediction revealed that rs9289981 could improve the binding affinity of *RBPJ* (**Figure 6E**). Previous studies have reported the important role of *RBPJ* in cancers (45, 46), indicating that rs9289981 may exert its function by influencing chromatin accessibility and regulating *CMSS1* expression.

### User-friendly data portal

**Figure 7:**
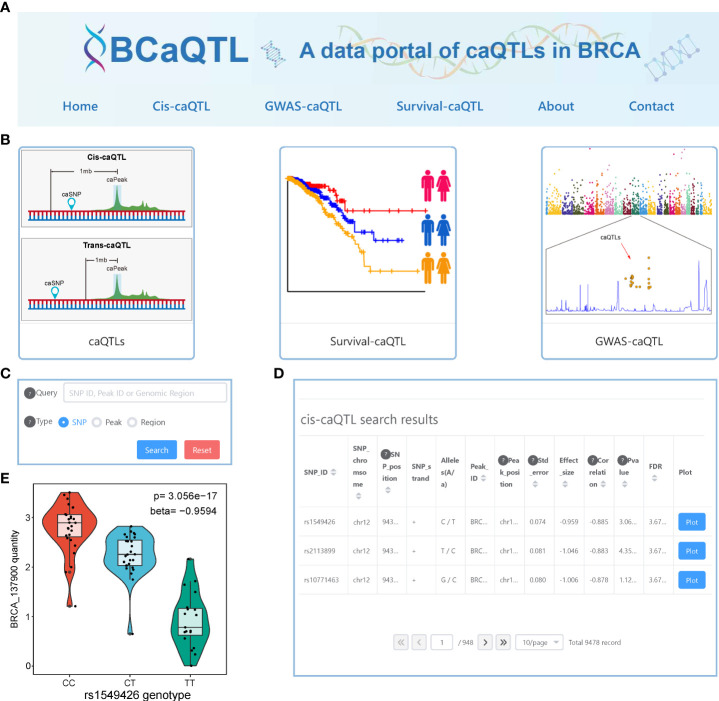
Interface of BCaQTL data portal. **(A)** Browser bar in BCaQTL. **(B)** Modules of cis-caQTL, GWAS caQTL, and survival caQTL. **(C)** The interface of the quick search panel. **(D)** An example of search results of the cis-caQTL dataset. **(E)** An example of the box plot shows the difference in chromatin accessibility between genotypes embedded in the search results.

To facilitate easy access to the relevant data, we developed a data portal with a user-friendly interface, BCaQTL (http://gong_lab.hzau.edu.cn/caqtl_database, **Figure 7A**), to store all our results for searching, browsing, and downloading. Except for the home page, the about page, and the contact page, we developed three modules for data retrieval (**Figure 7B**). On the cis-caQTL page, we provided a quick search (**Figure 7C**) where the users can quickly obtain information on caQTL SNPs (**Figure 7D**). For each record, a vector diagram of the boxplot is embedded (**Figure 7E**). On the survival-caQTL page, users can obtain information on the statistical result of survival analysis and the KM-curve plot. The GWAS-related caQTL page provides information on the caQTL SNPs, GWAS SNPs, and LD scores.

## Discussion

We performed a caQTL analysis in this study to link the genetic variants to chromatin accessibility. Prior to the caQTL mapping, we found that open chromatin regions can represent active regions in the genome. Specifically, open chromatin regions are enriched in active regulatory regions such as promoters and enhancers, and the binding sites of TFs are enriched in these open regions. More importantly, genes with an ATAC-peak overlapping the TSS usually have a higher expression than those without an ATAC-peak on the TSS site. Among the 23 human cancer types, we only identified cis-caQTLs in breast cancer, mainly ascribed to the small sample size in this study. It has been reported that the sample size can significantly affect QTL mapping, and the discovery of eQTLs and their related genes increases with sample size with no apparent saturation at the sample size of 300 (47), suggesting that more samples should be included to improve the quantity of the identification of caQTLs in the future studies.

The multi-omic data from the same individuals make it possible for us to investigate the cascading effect of genetic variation rather that overlap them. Using the eQTLs that came from the same collection of TCGA cancer donors, we investigated the relationship between chromatin accessibility and gene expression using causal inference. We found computational evidence for mediating effect in some of the SNP-peak-gene pairs. Those results could provide further insights into the regulatory mechanisms of eQTLs.

In GWAS analysis, due to genetic linkage disequilibrium, a cluster of SNPs is usually found in the region associated with a specific disease. SNP with the smallest P-value was typically chosen as the tag SNP, and the region around the SNP was defined as a risk region. However, the actual causal variants and the target genes in these risk loci and the underlying mechanisms remain largely unknown. In this study, we found that the variants altering chromatin accessibility are more likely to be located in the LD regions of GWAS tag SNPs rather than directly overlapping them. Our results indicated that caQTL SNPs instead of tag SNPs might act as the actual causal variants in breast cancer risk loci. In our study, we further identified the caQTLs that may affect TF binding, and these caQTLs are more likely to be causal variants in GWAS loci.

The dominant and recessive models are often used in GWAS because individuals with heterozygous genotypes sometimes have a similar phenotype to those with homozygous genotypes. With the observation that the caQTLs showed the same effect on survival time, we performed survival analysis using the dominant and the recessive models. As a result, we found more significant survival-related caQTLs than the additive model that classifies the individuals into three genotype groups.

In conclusion, we performed caQTL analyses in human cancer and identified thousands of functional SNPs, providing a new perspective for deciphering the effects of genetic variants in non-coding regions. Our BCaQTL database will be an important resource for genetic and epigenetic studies.

## Data availability statement

Publicly available datasets were analyzed in this study. This data can be found here: TCGA data portal (https://tcga-data.nci.nih.gov/tcga/).

## Author contributions

JG and KT conceived the idea and supervised the study. DW designed and performed the analysis. XW, GJ, JY, ZY, and WY collected the data. DW and YY developed the website. DW and XW searched the literature and wrote the manuscript. JG and XN revised the manuscript. All authors contributed to the article and approved the submitted version.

## Funding

This work was supported by the National Natural Science Foundation of China (31970644 to JG) and Huazhong Agricultural University Scientific & Technological Self-innovation Foundation (11041810351 to JG), and the Fundamental Research Funds for the Central University HZAU (Grant No. 2662017JC048 to XN).

## Conflict of interest

The authors declare that the research was conducted in the absence of any commercial or financial relationships that could be construed as a potential conflict of interest.

## Publisher’s note

All claims expressed in this article are solely those of the authors and do not necessarily represent those of their affiliated organizations, or those of the publisher, the editors and the reviewers. Any product that may be evaluated in this article, or claim that may be made by its manufacturer, is not guaranteed or endorsed by the publisher.

## Supplementary material

Supplementary Figure 1 Enrichment of ATAC-peaks in different chromatin states across 23 cancer types. The background peaks were genomic regions with the same width randomly selected.

Supplementary Figure 2 Differences in gene expression levels between genes with ATAC-peaks overlap with the TSS, and those without ATAC-peaks overlap with the TSS.
