# Supplementary figures and images for: Systematic analysis of the effects of genetic variants on chromatin accessibility to decipher functional variants in non-coding regions
Source: Front Oncol. 2022 Oct 18;12:1035855. doi: 10.3389/fonc.2022.1035855 (PMC9623183; doi:10.3389/fonc.2022.1035855)

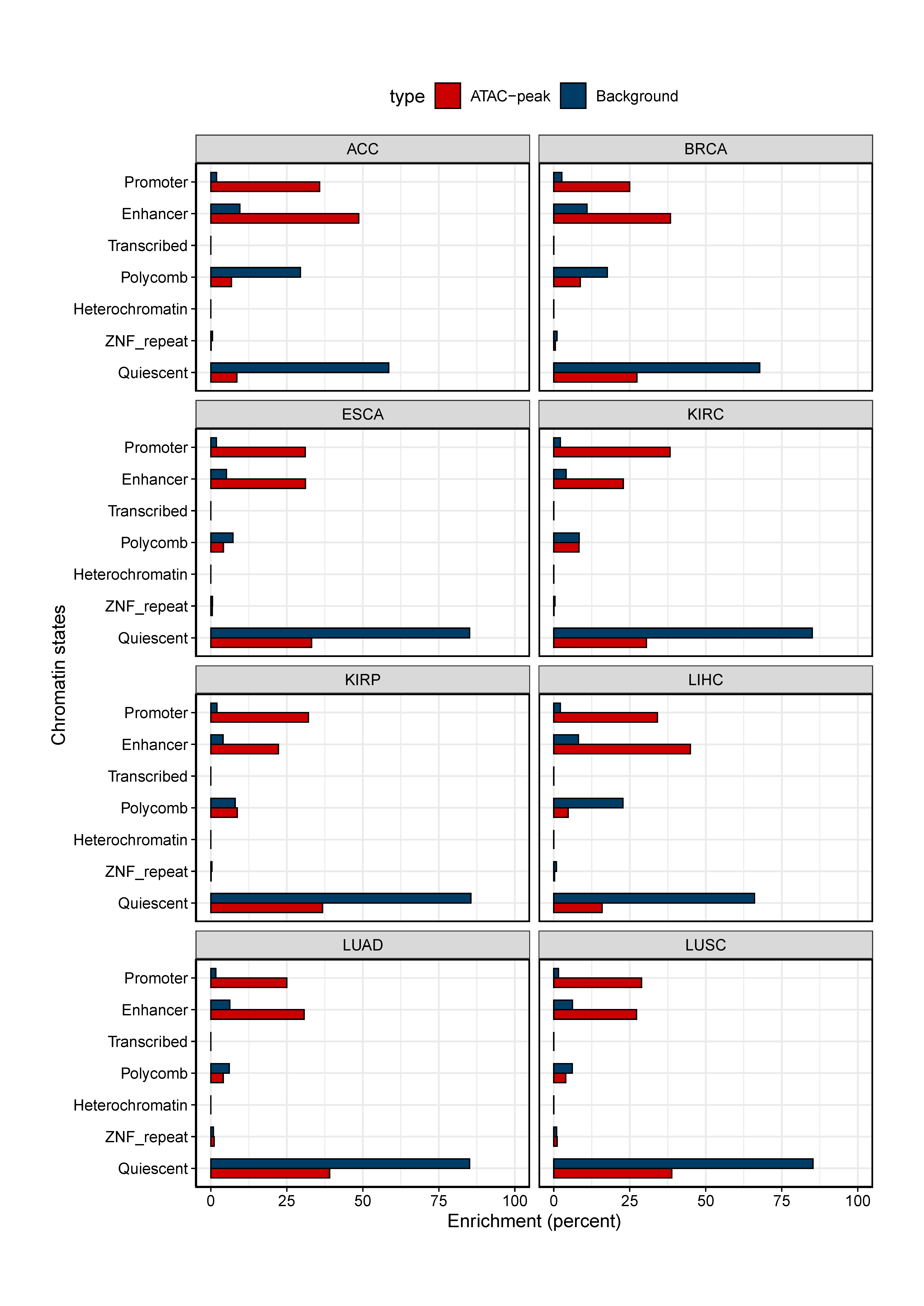

Supplement: Supplementary Figure 1 — Enrichment of ATAC-peaks in different chromatin states across 23 cancer types. The background peaks were genomic regions with the same width randomly selected. [file Image_1.tiff]

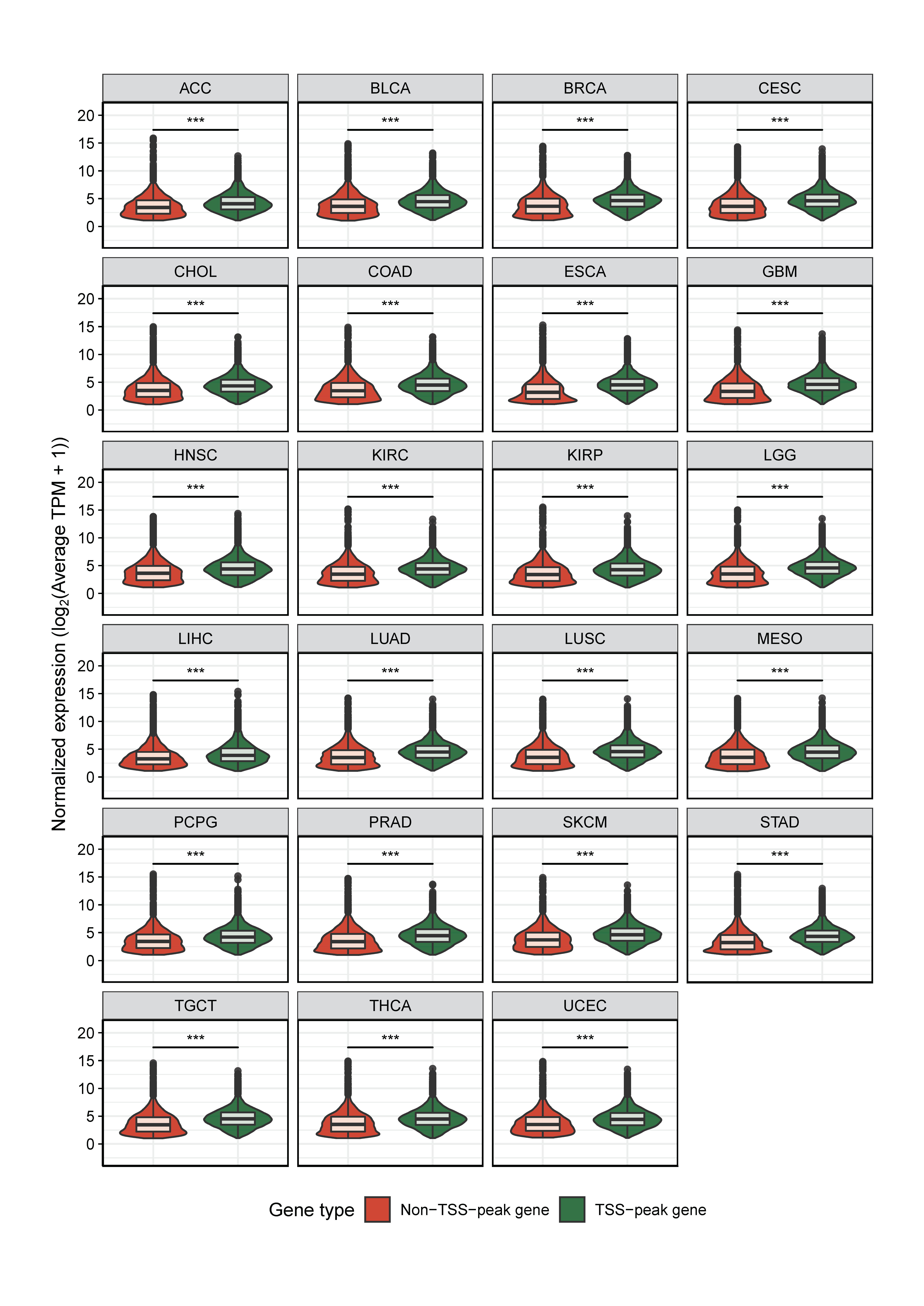

Supplement: Supplementary Figure 2 — Differences in gene expression levels between genes with ATAC-peaks overlap with the TSS, and those without ATAC-peaks overlap with the TSS. [file Image_2.tiff]
